# A cross-sectional observational study of the nutritional intake of UK primary school children from deprived and non-deprived backgrounds: implications for school breakfast schemes

**DOI:** 10.1186/s12966-015-0238-9

**Published:** 2015-06-25

**Authors:** Kim T Jenkins, David Benton, Katy Tapper, Simon Murphy, Laurence Moore

**Affiliations:** Department of Psychology, Swansea University, Swansea, SA2 8PP UK; Department of Psychology, City University London, London, EC1R 0JD UK; DECIPHer, School of Social Sciences, Cardiff University, Cardiff, CF10 3BD UK; MRC/CSO Social and Public Health Sciences Unit, Institute of Health and Wellbeing, University of Glasgow, Glasgow, G2 3QB UK

**Keywords:** Breakfast, Nutrition, Children, Deprivation, Free school breakfast scheme, Diet, Dietary recall interview

## Abstract

**Background:**

This study examined the nutritional intake of 9–11 year old children in Wales, UK, to assess the rationale for, and potential of, school breakfast initiatives. It also examined the possible unintended consequence of over consumption.

**Methods:**

The study employed a cross-sectional observational design within a randomized controlled trial of a free school breakfast programme. A total of 111 primary schools were randomly assigned to an intervention condition (in which a free school breakfast programme was implemented) or a control condition (in which implementation of the scheme was delayed). Sub-samples of children completed multiple-pass 24-hr dietary recall interviews at baseline (*n* = 581), and 12 months later (*n* = 582). Deprivation was assessed for each child in terms of whether or not they were entitled to free school meals.

**Results:**

Prior to the introduction of the programme, rates of breakfast skipping were low and there was little evidence of widespread nutritional deficiency. However, there was a subset of children who consumed inadequate levels of a range of vitamins and minerals and 29 % of children ate very little for breakfast (less than 100 kcal). Children that ate larger breakfasts, had higher daily intakes of all nutrients that were examined. Children from deprived backgrounds consumed significantly lower levels of several vitamins and minerals at breakfast. Following the introduction of the breakfast scheme in intervention schools, there was little difference in the nutritional quality of school versus home breakfasts (*n* = 35 and 211 respectively). Where children ate breakfast at both school and home (*n* = 33), their overall energy intake was higher, but not significantly so.

**Conclusions:**

Although the overall diet of this group of children was generally good prior to the breakfast scheme, the results suggest that such schemes could be beneficial for a subset of children who are poorly nourished and for those children who consume very little for breakfast.

**Trial registration:**

Current Controlled Trials ISRCTN18336527

## Background

Research suggests that breakfast makes an important contribution toward the diets of primary school children. For example, studies conducted in North America and Europe have found that children who eat breakfast tend to have higher intakes of vitamin A, vitamin C, riboflavin, calcium, zinc, iron and fibre [1]. Conversely, breakfast skipping has been linked to dietary inadequacy. For example, a study of African American children found that amongst those who skipped breakfast, substantial proportions consumed less than 50 % of the recommended daily intakes of a wide range of nutrients, including vitamin C, vitamin A, vitamin E, calcium and iron [2]. Similarly, research in New Zealand found that children who skipped breakfast were less likely to achieve the recommended intake of fruit and vegetables [3]. Additionally, a number of studies have linked breakfast skipping to an increased risk of overweight and obesity [1, 4, 5].

However, despite these advantages, breakfast consumption amongst children has declined [6–8], with some studies reporting that up to 20 % of primary age children regularly skip breakfast [4, 9]. Breakfast skipping may be particularly pronounced amongst children of lower socioeconomic status [10] that may in turn contribute to health inequalities.

It is within this context that a number of governments have set up school breakfast initiatives with both nutritional and educational aims [11]. However, little is known about their effects on diet since few rigorous evaluations have been conducted. One exception is a recent randomized controlled trial evaluation of a North American free school breakfast programme. Whilst this showed that the programme improved the nutritional quality of breakfast, it had no impact on rates of breakfast skipping or on dietary intake over a 24-hr period [12]. There has also been some concern that school breakfasts may inadvertently increase intake of saturated fat and contribute to obesity, particularly where children may be consuming two breakfasts [13, 14]. Consequently, there is a need to assess such unintended consequences.

However, given the diversity of populations at which breakfast programmes may be targeted, together with the range of foods that may be provided, it is difficult to draw any general conclusions about their potential effects. For example, whilst they may have considerable benefits where diet tends to be poor, they may have limited impact where children are already well-nourished. Breakfast programmes may also help reduce health inequalities even where their overall effect is minimal. For these reasons, examining the nutritional profile of a target population provides important contextual information in which to interpret the effects of a breakfast intervention.

The present study examines dietary data collected during the evaluation of the Welsh Assembly Primary School Free Breakfast Initiative. This programme was launched in 2004 with a view to improve children’s health and educational attainment, as well as reduce health inequalities. It was rolled out in such a way as to allow a cluster randomized controlled trial evaluation and included dietary questionnaires collected with over 4,000 children aged 9–11 years. These questionnaires enabled analyses of numbers of ‘healthy’ versus ‘unhealthy’ food items (e.g., fruit versus crisps) consumed at breakfast and throughout the day. The data indicated that the programme increased the number of breakfasts consumed at school versus home, and also increased the number of healthy food items consumed at breakfast [15]; these increases in consumption of healthy food items were largest in more deprived schools [16]. The data also showed that children in more deprived schools ate more ‘unhealthy’ and fewer ‘healthy’ items prior to the introduction of the programme [17].

The present paper examines additional dietary recall interview data collected during this evaluation both prior to, and following, the introduction of the breakfast scheme. Since the dietary recall interviews were considerably more labour intensive than the dietary questionnaires, they were conducted with a subset of children only and thus were not sufficiently powered to evaluate the effects of the breakfast programme. However, unlike the dietary questionnaire, the interview included questions about portion size and the data were of sufficient detail to estimate intake of specific nutrients. The present paper uses these data to provide a more detailed analysis of the nutritional intake of these children. The analyses aimed to explore the need for breakfast provision by examining: (1) The overall quality of these children’s diets, (2) breakfast quality amongst children from deprived versus non-deprived households, and (3) associations between breakfast size and overall diet quality, prior to the introduction of the breakfast programme. Whilst several studies have examined the relationship between breakfast skipping and nutritional intake [4, 18], few have explored relationships with breakfast size. This is important since even though rates of breakfast skipping may be relatively low amongst a particular population, this does not necessarily mean all children are consuming an adequate breakfast, and the size of their breakfast may be related to their overall diet.

Given that the breakfast programme changed the pattern of breakfast consumption across school and home [15], the analyses also examines data collected following the introduction of the scheme to (4) compare the quality of breakfasts eaten at home versus school, as well as (5) examine associations between the consumption of two (versus one) breakfasts and daily nutritional intake. These analyses should help provide more information about the nutritional content of school versus home breakfasts as well as address the question of whether consumption of two breakfasts could have a negative impact in terms of obesity.

## Methods

### Design

The study employed a repeated cross-sectional observational design embedded within a cluster randomised controlled trial, with the school as the unit of randomization [17].

### Participants

From 111 schools, 4,350 children aged 9–11 years were recruited at baseline and 4,472 at follow-up. Of the participating schools, 58 were located in ‘Communities First’ (less affluent) areas and 53 were located in ‘Non-Communities First’ (more affluent) areas. Schools were assigned to either the intervention group (the immediate implementation of the Free School Breakfast programme) or the control group (one year delay before implementation of the programme) using stratified block randomization, with strata defined by LEA, school size, free school meal entitlement, and Welsh or English as the language of tuition.

In each school 6–9 children were randomly selected to undertake the dietary recall interview. Interviews were conducted at two separate time points; baseline (during the academic years 2004/5 and 2005/6) and at follow-up 12 months later. Since children in Year 6 at baseline had moved to secondary school at follow-up, the study employed a repeated cross-sectional observational design, which meant that children who completed dietary recall interviews at baseline were not necessarily the same as those who completed them at follow-up. In total 1,322 children were interviewed although prior to analysis the data of 149 children were excluded for various reasons including: ill health, absence from school, vague or missing recall data or an inability to understand the procedure (e.g., learning disabilities or a poor comprehension or expression of the English language). After excluding children who did not eat breakfast (5 children at baseline and 5 at follow-up), 581 children (270 males, 311 females) were considered at baseline and 582 at follow-up (288 males, 294 females).

### The primary school free breakfast initiative

The Primary School Free Breakfast Initiative provided a meal before the commencement of classes, without any cost to the parent. Schools running the scheme were asked to give children the option of one item from each of the following four types of food: non-sugar coated breakfast cereal; bread; milk based drinks and milk products; fruit, including unsweetened fruit juice. In practice the majority of schools offered breakfast cereal with milk, toast and fruit juice and either fresh, dried or tinned fruit.

### Measures

#### Dietary recall interviews

Dietary recall interviews were conducted using a standardized protocol [19], a structured multiple-pass design. The first pass required the child to tell the researcher everything they had eaten or drunk on the morning of testing and the previous day. The opening question was; “After you got up this morning/yesterday morning, when was the first time that you had something to eat or drink?”, followed by the questions “What did you eat or drink at that time?” and “Did you eat or drink anything else at that time?” The same three questions were repeatedly asked until the child had recalled all the food and drink items consumed over the specified period. The researcher ended the first pass with the questions, “Is that the last time you had something to eat or drink?” and “Can you remember any other times you had something to eat or drink?” The second pass was identical to the first except in addition the researcher mentioned the recalled information to identify any items that had been forgotten or incorrectly recorded. A third pass required the children to give more details about the food and drink items consumed: for instance the name of the meal, where they had eaten the item and whether anything had been added to the meal (e.g., condiments, sugars, spreads). Details of the serving size were established using photographic visual aids [20, 21], from which the child selected the picture that most closely resembled their serving. A final pass reviewed all previously recalled information to confirm the accuracy of the record.

Breakfast was defined as all food and beverages consumed before the start of the school day (9 am). The data were analyzed using CompEat Nutritional Analysis Software (Nutrition Systems, Banbury, UK) that uses the McCance and Widdowson food tables [22]. Where information was not available nutritional information was obtained from food labels or other manufacturer’s data. The reliability of coding was established by comparing the results from pairs of coders that found typical inter-observer reliability in excess of 0.9. A nutritional profile was calculated both for breakfast and an entire 24-hr period. Where appropriate the intake was compared to the United Kingdom Dietary Reference Values [23].

#### Deprivation

Deprivation was assessed for each child in terms of whether or not they were entitled to free school meals (yes versus no). These data were obtained from the Secure Anonymised Information Linkage (SAIL) databank.

### Procedure

Informed consent was obtained from the school, opt-out consent was obtained from parents, and children were also informed that they were under no obligation to participate. Parents of fifteen children asked that their child be excluded. Ethical approval for the original trial was granted by the Cardiff University Social Science Ethics Committee. Ethical approval for further analysis of the dietary data was granted by the National Research Ethics Service.

The dietary recall interviews were conducted on a one-to-one basis in a quiet location in the school. The majority took place between 12.30 and 3.30 pm, though due to timetabling restrictions they were occasionally scheduled as early as 11 am. All interviews were audio recorded and 10 % of these audio-recordings were checked to ensure that each interviewer had adhered to the protocol.

### Statistical analysis

The nutritional data, both for breakfast and a 24-hr period were summarized and are reported as means and standard errors. Differences between children from deprived and non-deprived households were compared using t-tests. The effect of size of breakfast on total dietary intake was considered using one-way analysis of variance. Comparison of breakfast location (school versus home versus both) on dietary intake was also examined using one-way analysis of variance.

## Results

### Overall diet quality

Table 1 shows macro- and micro-nutrient intakes of boys and girls (*n* = 581) over a 24-hr period, prior to the introduction of the breakfast programme. For boys, in the cases of calcium, magnesium, iron, zinc, copper, selenium and iodine, average intake was greater than the reference nutrient intake (RNI) [23]. Similarly the average intake of vitamins A, B_1_, B_2_, B_6_, B_12_, C, nicotinic acid equivalents and folate exceeded the RNI. For sodium, the recommendation is that children aged 7 to 10 should not consume more than 2 grams a day. In our sample 65.4 % of boys and 59.5 % of girls had more than 2 grams.

**Table 1:** Twenty-four hr nutrient intakes, mean (SE), for boys and girls prior to the introduction of the breakfast programme

Nutrient	Reference Nutrient Intake^a^	Boys	Percent of boys less than 66 % of RNI	Girls	Percent of girls less than 66 % of RNI
		(*n* = 270)		(*n* = 311)	
Energy (Mj)		8.18 (0.20)		7.09 (0.15)	
Protein (g)	28.3	63.7 (1.8)	0.5 %	53.8 (1.3)	3 %
Fat (g)		75.3 (2.3)		65.3 (1.6)	
Carbohydrate (g)		268.6 (6.5)		232.9 (5.0)	
Fibre (g)		12.3 (0.4)		10.1 (0.3)	
Calcium (mg)	550	930.4 (36.6)	9.6 %	745.3 (23.3)	14 %
Phosphorus (mg)	450	1199.6 (31.9)	0 %	982.4 (22.0)	1.5 %
Magnesium (mg)	200	222.3 (5.8)	15 %	184.6 (4.0)	22.1 %
Sodium (mg)	1200	2688.2 (81.2)	0.5 %	2279.1 (56.2)	1.5 %
Potassium (mg)	2000	2353.7 (62.0)	10.6 %	2047.4 (50.5)	20.5 %
Chloride (mg)	1800	3932.5 (121.3)	1.6 %	3348.3 (80.4)	2.0 %
Iron (mg)	8.7	10.2 (0.35)	14.4 %	7.7 (0.2)	33.5 %
Zinc (mg)	7	7.2 (0.23)	22.9 %	5.9 (0.2)	35 %
Copper (mg)	0.7	1.0 (0.04)	10.6 %	0.8 (0.03)	17.5 %
Selenium (μg)	30	31.4 (1.3)	31.9 %	26.3 (1.1)	42.7 %
Iodine (μg)	110	137.5 (5.2)	25.5 %	113.5 (4.1)	31.5 %
Vitamin B_1_ (mg)	0.7	1.61 (0.05)	0.5 %	1.32 (0.04)	4 %
Vitamin B_2_ (mg)	1	1.66 (0.05)	12.8 %	1.30 (0.09)	17.1 %
Niacin (mg)	12	28.12 (0.9)	1.1 %	23.8 (0.63)	3.5 %
Vitamin B_6_ (mg)	1	2.04 (0.6)	1.1 %	1.73 (0.05)	6 %
Vitamin B_12_ (μg)	1	3.53 (0.15)	4.3 %	2.87 (0.11)	7 %
Folate (μg)	150	213.87 (6.6)	6.4 %	173.4 (5.1)	19 %
Vitamin C (mg)	30	105.16 (4.8)	5.9 %	99.9 (4.0)	7 %
Vitamin A (μg)	500	533 (32.4)	37.2 %	492 (22.7)	43.5 %
Vitamin D (μg)		2.22 (0.14)		1.81 (0.10)	
Vitamin E (mg)		6.64 (0.27)		6.09 (0.20)	

^a^Dietary Reference Values for Food Energy and Nutrients for the United Kingdom (1991). London: HMSO. The data are for children aged 7 to 10 years

Although for girls the average of some of these minerals exceeded the RNI, with magnesium (intake of 185 mg c.f. RNI of 200 mg), iron (7.75 mg c.f. 8.7 mg), zinc (5.52 mg c.f. 7.0 mg) and selenium (26.3 μg c.f. 30 μg) it was less. For vitamins the average intake exceeded the RNI with the exception of vitamin A where the average intake was 492 μg compared the RNI of 500 μg.

Using an intake of 66 % of the RNI as a point at which there are reasons for concern, in boys 14.5 % failed to achieve this value in the case of magnesium, 13.4 % with iron, 24 % with zinc, 32.9 % with selenium, 25.1 % with iodine, 10.2 % vitamin B_2_ and 39.9 % with vitamin A. There was, however, with all nutrients a small percentage whose intake was less than 66 % of the RNI, even when the group average was well in excess of 100 % of the RNI. For example, although the average intake of vitamin C was more than three times the RNI, 4.9 % of boys and 6.4 % of girls had an intake less than 66 % of the RNI.

### Breakfast quality amongst children from deprived versus non-deprived households

Of the 581 children interviewed at baseline, 232 were excluded because of missing free school meal data. Of the remaining 349 children, the nutritional profiles of those who did and did not qualify for free school meals because of a low family income are reported in Table 2. Intakes of vitamin B_1_, vitamin C and potassium were significantly lower in those qualifying for free school meals.

**Table 2:** Breakfast nutrient intakes, mean (SE), prior to the introduction of the breakfast programme, for children who did and did not qualify for free school meals

Nutrient	No free school meals (*n* = 262)	Free school meals (*n* = 87)
Energy (Mj)	4.85 (0.16)	4.74 (0.31)
Protein (g)	8.5 (0.3)	7.7 (0.5)
Fat (g)	7.4 (0.4)	7.2 (0.6)
Carbohydrate (g)	46.6 (1.5)	46.7 (3.1)
Fibre (g)	1.8 (0.1)	1.8 (0.2)
Calcium (mg)	246 (15.9)	258.1 (32.7)
Phosphorus (mg)	202.5 (8.8)	142.2 (14.6)
Magnesium (mg)	41.7 (2.1)	33.5 (3.4)
Sodium (mg)	330.5 (15.3)	248.4 (26.2)
^a^Potassium (mg)	371.8 (13.8)	326.2 (21.6)
Chloride (mg)	512.0 (23.5)	444.7 (39.7)
Iron (mg)	2.6 (0.2)	2.6 (0.1)
Zinc (mg)	1.1 (0.1)	1.0 (0.1)
Copper (mg)	0.1 (0.1)	0.1 (0.1)
Selenium (μg)	3.3 (0.1)	3.1 (0.5)
Iodine (μg)	37.4 (1.8)	32.4 (3.1)
^a^Vitamin B_1_ (mg)	0.4 (0.02)	0.4 (0.03)
Vitamin B_2_ (mg)	0.6 (0.03)	0.5 (0.05)
Niacin (mg)	5.5 (0.2)	5.1 (0.4)
Vitamin B_6_ (mg)	0.4 (0.02)	0.4 (0.05)
Vitamin B_12_ (μg)	0.7 (0.04)	0.7 (0.06)
Folate (μg)	62.6 (3.2)	58.2 (5.9)
^a^Vitamin C (mg)	33.3 (1.8)	17.7 (2.6)
Vitamin A (μg)	65.5 (3.4)	65.1 (7.6)
Vitamin D (μg)	0.2 (0.02)	0.1 (0.04)
Vitamin E (mg)	0.6 (0.05)	0.6 (0.07)

^a^Differed significantly, *p* < .05

### Associations between breakfast size and overall diet quality

Table 3 reports the influence of the size of breakfast (prior to the introduction of the programme) on what was eaten over 24 hrs (*n* = 581). These were examined in 100 kcal intervals since this was considered to best reflect the range of meals consumed. Only 4 % of children did not consume any breakfast, whereas 29 % ate less than 419Kj (100 kcal), 49 % ate between 419 and 837 Kj (100–200 kcal), 12 % between 837 and 1256 Kj (200–300 kcal) and 6 % more than 1256 Kj (300 kcal). The pattern of consumption was very clear: the more energy that on average was consumed at breakfast the higher the consumption of every nutrient over a 24-hr period.

**Table 3:** Associations between size of breakfast and mean (SE) 24-hr nutritional intake, prior to the introduction of the breakfast programme

Nutrient	Size of breakfast					Significance df = 4,604
	0 Kj	1–419 Kj	420–837 Kj	838–1256 Kj	>1256 Kj	
Energy (Mj)	5.84 (0.55)	6.09 (0.18)	7.60 (0.14)	9.69 (0.28)	11.58 (0.81)	F = 50.7 *p* < 0.001
Protein (g)	49 (4.7)	47 (19.1)	59 (1.3)	72 (2.7)	91 (8.4)	F = 32.6 *p* < 0.001
Fat (g)	53 (4.7)	58 (2.2)	70 (1.6)	87 (3.0)	111 (10.5)	F = 29.7 *p* < 0.001
Carbohydrate (g)	180 (15.2)	197 (5.6)	252 (4.6)	327 (11.0)	369 (24.3)	F = 53.5 *p* < 0.001
Fibre (g)	7.7 (0.6)	9.1 (0.3)	10.8 (0.3)	14.7 (0.7)	18.3 (1.7)	F = 35.2 *p* < 0.001
Calcium (mg)	508 (79)	586 (22)	852 (24)	1031 (44)	1640 (189)	F = 47.7 *p* < 0.001
Phosphorus (mg)	836 (75)	833 (25)	1093 (23)	1403 (48)	1706 (126)	F = 51.2 *p* < 0.001
Magnesium (mg)	150 (13.2)	159 (4.5)	201 (3.9)	265 (9.4)	325 (22.7)	F = 57.4 *p* < 0.001
Sodium (mg)	2040 (215)	2055 (72)	2469 (60)	3061 (134)	3523 (348)	F = 20.1 *p* < 0.001
Potassium (mg)	1798 (161)	1798 (58)	2195 (49)	2846 (116)	2921 (249)	F = 25.5 *p* < 0.001
Chloride (mg)	2824 (325)	3031 (108)	3629 (88)	4540 (199)	4990 (491)	F = 19.1 *p* < 0.001
Iron (mg)	6.1 (0.7)	6.5 (0.2)	9.0 (0.2)	11.1 (0.5)	16.9 (1.9)	F = 54.4 *p* < 0.001
Zinc (mg)	5.4 (0.6)	5.2 (0.2)	6.5 (0.2)	8.2 (0.4)	10.5 (0.8)	F = 29.2 *p* < 0.001
Copper (mg)	0.7 (0.05)	0.7 (0.04)	0.9 (0.03)	1.1 (0.07)	1.4 (0.13)	F = 11.8 *p* < 0.001
Selenium (μg)	24 (3.3)	24 (1.4)	28 (1.0)	36 (2.7)	45 (4.9)	F = 12.3 *p* < 0.001
Iodine (μg)	86 (13)	89 (4)	137 (5)	153 (9)	163 (16)	F = 18.6 *p* < 0.001
Vitamin B_1_ (mg)	1.1 (0.13)	1.1 (0.04)	1.5 (0.04)	1.8 (0.07)	2.2 (0.20)	F = 24.1 *p* < 0.001
Vitamin B_2_ (mg)	0.9 (0.16)	1.0 (0.03)	1.6 (0.04)	1.9 (0.08)	2.4 (0.23)	F = 42.4 *p* < 0.001
Niacin (mg)	20 (2.2)	20 (0.7)	26 (0.06)	33 (1.3)	40 (3.1)	F = 36.7 *p* < 0.001
Vitamin B_6_ (mg)	1.4 (0.15)	1.4 (0.05)	1.9 (0.05)	2.3 (0.10)	2.9 (0.28)	F = 31.6 *p* < 0.001
Vitamin B_12_ (μg)	2.5 (0.37)	2.4 (0.13)	3.3 (0.12)	4.0 (0.26)	4.9 (0.51)	F = 16.3 *p* < 0.001
Folate (μg)	137 (20)	144 (5)	197 (5)	246 (12)	305 (29)	F = 34.1 *p* < 0.001
Vitamin C (mg)	87 (17)	84 (5)	104 (4)	135 (10)	122 (11)	F = 7.7 *p* < 0.001
Vitamin A (μg)	357 (67)	430 (28)	514 (24)	687 (58)	768 (159)	F = 7.5 *p* < 0.001
Vitamin D (μg)	1.5 (0.25)	1.5 (0.11)	2.0 (0.11)	2.6 (0.25)	3.3 (0.53)	F = 8.7 *p* < 0.001
Vitamin E (mg)	4.7 (0.56)	5.3 (0.56)	6.2 (0.21)	8.5 (0.53)	8.8 (0.80)	F = 14.1 *p* < 0.001

### Quality of breakfasts consumed at home versus at school

In schools where the breakfast programme was introduced, of the 279 children who ate breakfast, 35 (13 %) ate it at school only, and 33 (12 %) ate it at both home and school. Table 4 lists the nutritional profile of the breakfast of those eating at home, at school and at both. There were significant differences in the energy, fat, and carbohydrate intakes. Unsurprisingly those eating two meals had an intake that was significantly greater than those eating only at home, in the cases of energy, fat and carbohydrate. Intake of potassium, chloride, selenium, folate, and vitamin C also differed with style of breakfast. In most cases the nature of the meal did not differ between school and home although significantly greater amounts of carbohydrate were consumed as part of a school rather than home-based breakfast.

**Table 4:** Nutritional composition of breakfasts consumed at home, at school and at both home and school, following the introduction of the breakfast programme

Nutrient intake at breakfast	Breakfast at home		Breakfast at school		Breakfast at school and home		F values d.f.
	(*n* = 211)		(*n* = 35)		(*n* = 33)		(2,276)
	Mean	se	Mean	se	Mean	se	
Energy (Mj)	1.10^a^	0.05	1.37	0.11	1.70^a^	0.15	10.70, *p* < 0.001
Protein (g)	8.8	0.47	9.0	1.12	11.1	1.13	
Fat (g)	7.3^a^	0.47	7.2	1.06	11.0^a^	1.36	3.99, *p* < 0.02
Carbohydrate (g)	48.7^a,b^	1.81	59.8^a^	4.62	69.7^b^	6.06	12.62, *p* < 0.001
Fibre (g)	2.07	0.15	3.29	0.46	3.55	0.44	8.37, *p* < 0.001
Calcium (mg)	258.4	19.9	235.6	29.8	309.5	42.4	
Phosphorus (mg)	203.5	11.5	196.1	27.0	246.0	26.0	
Magnesium (mg)	42.6	2.5	46.8	5.9	59.1	6.1	
Sodium (mg)	338.4	23.9	382.1	37.7	484.6	55.6	
Potassium (mg)	394.3^a^	18.4	446.7	41.5	613.6^a^	64.8	8.78, *p* < 0.001
Chloride (mg)	524.9^a^	38.0	629.6	59.6	780.9^a^	89.7	3.48, *p* < 0.03
Iron (mg)	2.6	0.20	2.4	0.33	3.2	0.38	
Zinc (mg)	1.11	0.07	1.08	0.15	1.31	0.16	
Copper (mg)	0.12	0.01	0.13	0.01	0.17	0.03	
Selenium (μg)	3.2^a,b^	0.19	5.1^a^	0.99	5.62^b^	0.82	9.49, *p* < 0.001
Iodine (μg)	38.2	2.5	33.1	5.5	45.8	7.4	
Vitamin B_1_ (mg)	0.37	0.02	0.36	0.04	0.51	0.05	
Vitamin B_2_ (mg)	0.58	0.03	0.47	0.08	0.66	0.08	
Niacin (mg)	5.3	0.28	5.4	0.74	6.7	0.76	
Vitamin B_6_ (mg)	0.42	0.03	0.31	0.05	0.54	0.08	
Vitamin B_12_ (μg)	0.75	0.05	0.51	0.09	0.77	0.14	
Folate (μg)	59.9^a^	3.7	67.0	10.08	92.8^a^	11.0	4.94, *p* < 0.008
Vitamin C (mg)	25.7^a^	2.5	32.9	5.2	53.8^a^	8.6	7.85, *p* < 0.001
Vitamin A (μg)	71.0	6.6	74.2	11.5	109.6	13.8	
Vitamin D (μg)	0.16	0.03	0.18	0.07	0.22	0.06	
Vitamin E (mg)	0.62	0.06	0.50	0.08	0.75	0.08	

Note. The data are means and standard errors. Where there were significant differences the F values are listed in the right-hand column. Lower case letters indicate pairs of means that differed significantly from each other at least at the *p* < 0.01 level, as indicated by Scheffe’s test

### Associations between consumption of two (versus one) breakfasts and daily nutritional intake

Table 5 shows nutritional intake over 24 hrs among children consuming one breakfast (at home or school) or two breakfasts (i.e. at home and school; *n* = 279, analysis was restricted to schools where the breakfast programme was introduced). Total energy intake over the 24-hr period was higher for children who had eaten two breakfasts, but this difference did not reach statistical significance. However, there was a significantly greater consumption of carbohydrate, vitamin B1, folate, and vitamin C amongst those who ate two breakfasts.

**Table 5:** Nutritional intake over 24 hrs among children who consumed breakfast at home, at school, or at both home and school, following the introduction of the breakfast programme

Nutrient intake over 24 hrs	Breakfast at home		Breakfast at school		Breakfast at school and home		F values d.f.
	(*n* = 211)		(*n* = 35)		(*n* = 33)		(2,276)
	Mean	se	Mean	se	Mean	se	
Energy (Mj)	7.57	0.20	7.63	0.50	8.80	0.60	
Protein (g)	59.4	1.7	62.1	4.7	68.3	5.5	
Fat (g)	68.7	2.3	64.5	5.9	75.8	6.3	
Carbohydrate (g)	253.6^a^	6.5	259.5	15.6	308.8^a^	19.2	4.68, *p* < 0.01
Fibre (g)	14.5	0.53	15.8	1.44	18.2	1.52	3.22, *p* < 0.04
Calcium (mg)	885.5	39.1	779.8	57.4	926.6	98.1	
Phosphorus (mg)	1110.8	32.0	1110.8	75.9	1233.8	107.0	
Magnesium (mg)	212.0	6.1	214.8	15.1	245.5	20.8	
Sodium (mg)	2395.1	72.0	2428.9	198.2	2794.8	255.0	
Potassium (mg)	2280.7	66.7	2550.3	235.3	2735.6	228.8	31.3, *p* < 0.05
Chloride (mg)	3565.3	119.4	3495.2	283.6	4174.7	359.7	
Iron (mg)	9.2	0.34	8.8	0.59	9.2	0.29	
Zinc (mg)	6.7	0.23	6.5	0.5	7.2	0.63	
Copper (mg)	0.96	0.06	1.11	0.20	1.00	0.09	
Selenium (μg)	32.9	1.68	39.9	5.8	34.6	4.4	
Iodine (μg)	142.4	6.1	129.7	12.4	151.9	20.2	
Vitamin B_1_ (mg)	1.51^a^	0.05	1.36^b^	0.11	1.94^a,b^	0.19	5.09, *p* < 0.01
Vitamin B_2_ (mg)	1.54	0.06	1.39	0.11	1.65	0.19	
Niacin (mg)	26.5	0.79	28.9	2.57	30.1	2.49	
Vitamin B_6_ (mg)	2.02	0.69	1.96	0.17	2.49	0.22	3.15, *p* < 0.04
Vitamin B_12_ (μg)	3.36	0.16	3.51	0.51	3.13	0.42	
Folate (μg)	204.3^a^	6.8	213.9	19.8	262.2^a^	26.6	4.04, *p* < 0.02
Vitamin C (mg)	112.8^a^	5.5	125.0	17.6	153.6^a^	119.6	3.23, *p* < 0.04
Vitamin A (μg)	586.6	41.2	586.4	91.5	824.4	119.3	
Vitamin D (μg)	2.21	0.14	1.96	0.37	2.37	0.39	
Vitamin E (mg)	6.29	0.15	6.06	0.74	6.74	0.74	

Note. The data are means and standard errors. Where there were significant differences the F values are listed in the right-hand column. Lower case letters indicate pairs of means that differed significantly from each other at least at the *p* < 0.02 level, as indicated by Scheffe’s test

## Discussion

The results showed that, prior to the introduction of the breakfast programme, the overall quality of children’s diets was good. Of the 20 vitamins and minerals examined (excluding sodium), boys’ average daily intake exceeded recommended levels in all instances. For girls, average intake fell below recommended levels for nearly a quarter of the nutrients (magnesium, iron, zinc, selenium, vitamin A), but in no instance did average intake fall below 84 % of recommended levels.

However, average intakes can mask individual differences in diet quality. The results also showed that between 10 and 37 % of boys had inadequate intakes of a range of nutrients, namely magnesium, potassium, iron, zinc, copper, selenium, iodine, vitamin B_2_ and vitamin A. Similarly for girls, between 10 and 44 % had inadequate intakes of calcium, magnesium, potassium, iron, zinc, copper, selenium, iodine, vitamin B_2_, folate and vitamin A. Thus whilst average nutrient intake at the population level was generally good, there was a sizable proportion of children who were failing to consume sufficient quantities of a range of different nutrients. This finding is in line with other research in the UK that has shown that amongst children of this age there tends to be a subset that are poorly nourished [24]. Thus it is possible that a breakfast scheme could have a minimal impact on average diet, but could still make an important contribution to reducing health inequalities, particularly if it was able to target poorly nourished children.

In terms of the nutritional content of breakfasts, the results showed that children from more deprived backgrounds consumed significantly lower levels of potassium, vitamin B_1_ and vitamin C at breakfast. Previous analysis of the association between deprivation and breakfast with this sample has been inconsistent; whilst results showed that children at schools located in more compared to less deprived areas reported consuming fewer ‘healthy’ items for breakfast (i.e. fruit, bread, cereal, milk) [10], when deprivation was assessed at the household level this association disappeared [16]. However, both these analyses employed a dietary questionnaire that simply recorded the number of items consumed. The dietary recall interview employed in the present analysis also recorded portion size and amount eaten. It therefore seems plausible that whilst there may have been little difference in the number of healthy foods children from deprived versus non-deprived backgrounds were presented with, those from deprived backgrounds may have eaten smaller quantities. This interpretation is supported by the fact that children from deprived households reported eating more unhealthy food items at breakfast [16] that may have displaced consumption of more healthy items. It is also consistent with the fact that typical breakfast foods such as fruit, bread, and yoghurt tend to be high in potassium, vitamin B_1_ and vitamin C. The results highlight the importance of taking account of portion size and amount consumed when assessing children’s diet. The results are also in line with other research showing lower intake of a range of nutrients among children living in households receiving state benefits [24]. Again the findings illustrate the way in which a school breakfast programme may potentially help reduce health inequalities by improving the nutritional content of breakfast consumed by children from more deprived backgrounds.

Breakfast skipping in the current study was 4 %. This figure is lower than 15–20 % reported with US children of this age [4, 9, 25] but in line with recent research conducted in the UK that found that 6 % of primary school children skipped breakfast [26]. However, the results also showed that where breakfast was consumed its size varied markedly. In particular, 29 % of children ate less than 419Kj (100 kcal). This finding illustrates the importance of considering size of breakfast as well as breakfast skipping when considering the diets of school children. Indeed, in the current sample breakfast size clearly made an important contribution to overall nutritional intake, with children who ate more substantial breakfasts having higher daily intakes of all nutrients that were examined. This supports other studies that have highlighted the important contribution breakfast makes to diet [1, 2]. Additionally, other work has shown that eating a small breakfast may contribute to poorer academic functioning. For example, amongst primary school children, eating a small breakfast (average 61 kcal) was associated with less on task behaviour in the classroom during the morning [27]. Thus eating a more substantial breakfast may be desirable from both nutritional and educational perspectives.

A comparison of breakfasts eaten at home versus school, following the introduction of the breakfast programme, showed little difference in nutritional content, with the exceptions of selenium and carbohydrate which were significantly higher in school breakfasts. However, it should be noted that since there were only 35 children in the present study eating school breakfast only, it is difficult to draw any firm conclusions from these data. The randomized controlled trial evaluation of this particular breakfast programme showed a greater consumption of healthy food items in intervention schools following the introduction of the programme, according to dietary questionnaires [15].

The current study also showed that of those who ate breakfast at school, 49 % had already eaten at home. This is a potential cause for concern since the increased energy intake could predispose to obesity [14]. In the current study analysis of total energy intake showed no significant difference between those who ate two breakfasts and those who ate one, though average levels were higher in those consuming two breakfasts. However, again it is important to note the relatively small number of children consuming breakfasts at both school and home (*n* = 33) as well as the fact that these findings may not generalize to other breakfast programmes where different types of foods are provided [13, 28, 29].

In conclusion, the current study found little evidence for breakfast skipping or nutritional deficiency amongst the target population as a whole, prior to the introduction of the Welsh Assembly Government Primary School Free Breakfast Initiative. However, there was a subset of children who consumed inadequate levels of a range of vitamins and minerals, and although few children skipped breakfast, a sizable proportion (29 %) ate very little for breakfast (less then 100 kcal). This may impact upon the overall quality of their diet since there was a clear association between size of breakfast and total daily nutrient intake; those who consumed larger breakfasts had more nutritious diets overall. The results also showed that deprivation was associated with a significantly lower intake of several vitamins and minerals at breakfast. Upon introduction of the free school breakfast scheme there was little evidence to indicate significant differences in the nutritional quality of school versus home breakfasts, though the small number of children consuming only a school breakfast (*n* = 35) inevitably limits the power of this analysis. In terms of unintended consequences, eating breakfast at both school and home did not result in significantly higher total daily energy intake, though again the small numbers limits the power of this analysis. Overall, the results highlight the need for free breakfast schemes amongst a significant subgroup in the population. The barriers to the take up of targeted means tested interventions for such groups, highlights the importance of universal breakfasts schemes for reaching the most disadvantaged. However, in order to maximize their effects, breakfast schemes need to be used by children who need them most; children from deprived households and those who eat little or nothing for breakfast.
